# Neurodevelopmental Outcome at Corrected Age of 2 Years among Children Born Preterm with Operative Vaginal Delivery: A Population-Based Study (LIFT Cohort)

**DOI:** 10.3390/jcm12154970

**Published:** 2023-07-28

**Authors:** Guillaume Ducarme, Victoria Fosse, Valérie Rouger, Norbert Winer, Cyril Flamant, Marion Olivier

**Affiliations:** 1 Department of Obstetrics and Gynecology, Centre Hospitalier Departemental, 85000 La Roche-sur-Yon, France; victoriacasagrande@hotmail.fr; 2 Department of Obstetrics and Gynecology, Centre Hospitalier Universitaire de Nantes, 44000 Nantes, France; norbert.winer@chu-nantes.fr; 3 Loire Infant Follow-Up Team (LIFT) Network, 44000 Nantes, France; valerie.bureau@chu-nantes.fr (V.R.); marion.olivier@reseau-naissance.fr (M.O.); 4 Department of Neonatalogy, Centre Hospitalier Universitaire de Nantes, 44000 Nantes, France; cyril.flamant@chu-nantes.fr

**Keywords:** long-term, mode of delivery, neurodevelopmental outcome, operative vaginal delivery, preterm birth

## Abstract

The aim of the study was to determine whether operative vaginal delivery (OVD) was associated with non-optimal neurocognitive development at the corrected age of 2 years for preterm singletons using the Loire Infant Follow-up Team (LIFT) longitudinal cohort, a French regional perinatal network and prospective, population-based cohort of preterm infants. For this study, we included women with cephalic singletons and planned vaginal delivery from 24 to 34 weeks’ gestation between 2006 and 2016. The main exposure was the mode of delivery (spontaneous vaginal delivery (SVD), OVD, and cesarean delivery (CS) during labor). The primary outcome was non-optimal neurodevelopmental outcome at the corrected age of 2 years assessed by a physical examination, a neuropsychological test, and/or a parental questionnaire. Secondary outcomes were survival at discharge and survival at discharge without morbidity. We used the multivariate logistic regression and propensity score methods to compare outcomes associated with OVD. The study included 1934 infants born preterm: 1384 (71.6%) with SVD, 87 (4.5%) with OVD, and 463 (23.9%) with CS. Neonates with SVD, OVD, and CS did not differ in survival (97.0%, 97.7%, and 97.8%, respectively; *p* = 0.79) or in survival without morbidity (82.8%, 86.2%, and 82.7%, respectively; *p* = 0.71). In survived infants, 1578 (81.6%) were evaluated at age two: 279 (17.7%) were considered to have a non-optimal neurodevelopmental outcome (18.3% after SVD, 18.0% after OVD, and 15.9% after CS; *p* = 0.57). Propensity score analysis showed that OVD was not associated with non-optimal neurocognitive development at age two, with an adjusted odds ratio (aOR) of 0.86 and a 95% confidence interval (95% CI) of 0.47–1.69, compared with SVD; and an aOR of 0.76 and a 95% CI of 0.31–1.8, compared with CS. Operative vaginal delivery was not associated with non-optimal neurocognitive development at 2 years of corrected age for preterm singletons.

## 1. Introduction

Worldwide, 11% of live births occur before 37 completed weeks of gestation (WG). About 85% of these births are moderate to late preterm babies (32–36 WG), 10% are very preterm babies (28–31 WG) and 5% are extremely preterm babies (<28 WG) [1]. Among preterm births, the mode of delivery of premature infants remains a major concern for children with failed randomized control trials [2] or biased retrospective studies [3]. In cases of cephalic presentation and planned vaginal delivery at less than 37 WG, no mode of delivery (spontaneous vaginal delivery (SVD), operative vaginal delivery (OVD), or cesarean delivery during labor (CS)) has demonstrated superiority in terms of maternal and/or neonatal morbidity and/or long-term neurocognitive development for these children. In large population-based cohorts of births before 34 WG, rates of OVD were rarely performed and reported rates were around 5% [4,5,6]. Jointly, guidelines from the Society for Maternal-Fetal Medicine and the American College of Obstetricians and Gynecologists recommended to diminish the first CD rate but did not consider the specific cases of premature labor [7], whereas the French National College of Obstetricians and Gynecologists considered that the available data on better neonatal prognosis did not justify a strong indication of elective CS, which is associated with a potential increased maternal morbidity [8]. Data about the long-term safety of OVD in preterm births in the literature are rather poor [2,3,4,5,6], which completely justifies collecting more robust prospective data from a large cohort about the long-term neurocognitive development of the newborn. Scientific evidence about the safety of OVD in these specific cases may greatly interest obstetricians who have no major and robust guidelines, even though other various perinatal problems related to preterm birth are more related to the prognosis of the newborn than the mode of delivery itself.

Therefore, the purpose of this study was to evaluate the association between OVD and non-optimal neurocognitive development at 2 years of corrected age among children born preterm at less than 34 WG using matching propensity scores to ensure comparability of the study groups.

## 2. Materials and Methods

### 2.1. Study Design and Patients

This is a secondary analysis of prospective data from the Loire Infant Follow-up Team (LIFT) cohort. The LIFT cohort is a French regional population-based cohort that includes all surviving preterm infants born at less than 34 WG since March 2003 [9]. As described in detail [9], a network of about 180 trained physicians from the Pays-de-Loire region monitors children in a standardized manner at 3, 9, 12, 18, and 24 months of corrected age; 36, 48, and 60 months of age; and, since 2013, also at 84 months of age. All included children in the LIFT cohort throughout the study period were cared for by different obstetric and neonatology teams who belong to the same perinatal network without any change in obstetric management, and especially the need for obstetric intervention at birth (operative vaginal delivery, cesarean delivery, etc.). Infants with congenital anomalies or genetic syndrome and infants whose parents declined their inclusion in the LIFT follow-up program were excluded. Multiple pregnancies, intrauterine growth restriction, cesarean section before labor, breech presentation, and children born alive but having died in the delivery room or in intensive care were also excluded. Clinical data (obstetric and neonatal) were collected prospectively for all preterm infants enrolled in the LIFT network.

For this study, we only included women with cephalic singletons and planned vaginal deliveries at 24–34 WG between 1 January 2006 and 31 December 2016. Three groups were established based on mode of delivery: SVD, OVD, and CS during labor performed for standard obstetrical indications. Operative vaginal deliveries were assisted vaginal deliveries of neonates using forceps, vacuum, or Thierry’s spatulas.

Before inclusion in the LIFT cohort, written informed consent was obtained from both of the child’s parents before the infants were included in the LIFT cohort. The LIFT cohort is registered with the French data protection authority in clinical research (*Commission Nationale de l’Informatique et des Libertés*, No. 851117), and it received a favorable assessment from the relevant ethics committee.

### 2.2. Data Collection

Maternal demographic characteristics collected were maternal age, pre-pregnancy body mass index (BMI, calculated as weight (kg)/[height (m)]^2^, based on height and the first weight noted in the obstetric record), medical history (i.e., previous diabetes, previous hypertension), type of conception (spontaneous or using assisted reproductive technology), tobacco use during pregnancy, and maternal and paternal socioeconomic status at the beginning of the pregnancy.

The pregnancy and labor characteristics collected were threatened preterm birth, gestational diabetes mellitus, corticosteroid treatment during pregnancy, magnesium sulfate during pregnancy, preterm premature rupture of membranes (PPROM), chorioamnionitis (confirmed by placental histology), induction of labor (IOL), gestational age at delivery, mode of delivery (SVD, OVD, or CS during labor), and instrument used (forceps, vacuum, or Thierry’s spatulas) for OVD.

Neonatal data included the particular level of neonatal care of the maternity unit (with a neonatal intensive care unit (NICU) or not), birth weight, height and head circumference, gender, 5-min Apgar score, and neonatal complications in the NICU: intraventricular hemorrhage (IVH), periventricular leukomalacia (PVL), bronchopulmonary dysplasia (BPD, defined by oxygen therapy after 36 WG), persistence of the ductus arteriosus with necessary medical and/or surgical treatment, and necrotizing enterocolitis (NEC). All preterm infants were evaluated by cranial ultrasonography in the NICU in order to detect neurological complications. An electroencephalogram and cerebral MRI were performed on clinical signs and/or systematically depending on the severity of the prematurity.

At 2 years of corrected age, a validated French version of the Ages & Stages Questionnaire (ASQ) was reported by the parents to assess neurodevelopmental status [10,11], combined with a clinical examination by an experienced pediatrician. The presence of cerebral palsy was determined, defined as chronic, non-progressive impairment of motor skills, posture, balance, coordination, tone, or reflexes. The presence of sensory deficits was analyzed: visual (need for corrected lenses or mono/bilateral blindness) and auditory (need for hearing aids or mono/bilateral deafness). Motor function was considered to be non-optimal when cerebral palsy was present or when the clinical examination revealed neurological signs of abnormal muscle tone (stretching of the sural triceps muscle and/or imbalance of passive axial tone with predominance over the extensors) during voluntary walking. At the end of the clinical examination, the child was classified into one of three groups: “normal”, “intermediate” or “abnormal”. Children present in the “intermediate” or “abnormal” categories were considered to have non-optimal neurocognitive development. The ASQ considered 5 domains: fine motor skills, gross motor skills, communication and language, individual skills, and problem solving. For each item, parents indicated ‘‘yes’’ (10 points), ‘‘sometimes’’ (5 points), or ‘‘not yet’’ (0 point) to describe their child’s ability to perform a task. The maximum overall ASQ score was 300. Taking into consideration the results of large studies about non-optimal neurocognitive development at 2 years of age [11,12,13], at the end of the clinical examination, the child was thus considered as having non-optimal neurocognitive development at 2 years of corrected age in the case of a non-optimal clinical examination and/or ASQ below 185.

### 2.3. Endpoints

The primary outcome was non-optimal neurocognitive development at 2 years of corrected age that included children with non-optimal clinical examination and/or non-optimal psychomotor development according to the ASQ scale. Children who did not have a documented physical examination or psychomotor assessment were considered as “lost to follow-up”.

Secondary outcomes were neonatal survival at discharge and survival at discharge without morbidity. Neonatal morbidity was a composite variable, defined by at least one of the following criteria: severe IVH grade 3 or 4, PVL, BPD, persistence of the ductus arteriosus with necessary medical and/or surgical treatment, and NEC.

### 2.4. Exposure Variable

In this study, the neonatal and long-term characteristics of the children were compared according to the mode of delivery (SVD, OVD, and CS during labor).

### 2.5. Adjustment Variables

All variables that might influence neurocognitive development at 2 years of corrected age for preterm singletons were used as adjustment variables. Thus, the maternal, obstetric, and neonatal characteristics used in this study as adjustment variables included maternal socioeconomic status, gestational age at birth (32 to 34, 28 to 31, 25 to 27 WG), gender, birth weight expressed in standard deviations by a Z-score (<−1; −1 to 0; 0 to 1; and >1 SD), rupture of membranes longer than 24 h, pediatric procedures carried out immediately in the delivery room, and periventricular leukomalacia.

### 2.6. Statistical Analysis

Continuous variables were described by their means and standard deviations and compared between groups by a Wilcoxon test. Categorical variables were described by proportions and compared by χ^2^ tests (or Fisher’s exact tests for small numbers). We used logistic regression models, with multiple adjustments, to estimate the crude and adjusted associations between OVD and neurocognitive development at 2 years of corrected age for preterm singletons.

The propensity score analyses were performed as sensitivity analyses to confirm the results of the multivariate logistic regressions. The propensity score was based on a logistic regression model that included all the covariates that were significantly differently distributed according to whether the mode of delivery was OVD or CS delivery. R software was used for all analyses. *p* values <0.05 were considered to be statistically significant.

## 3. Results

**Figure 1:**
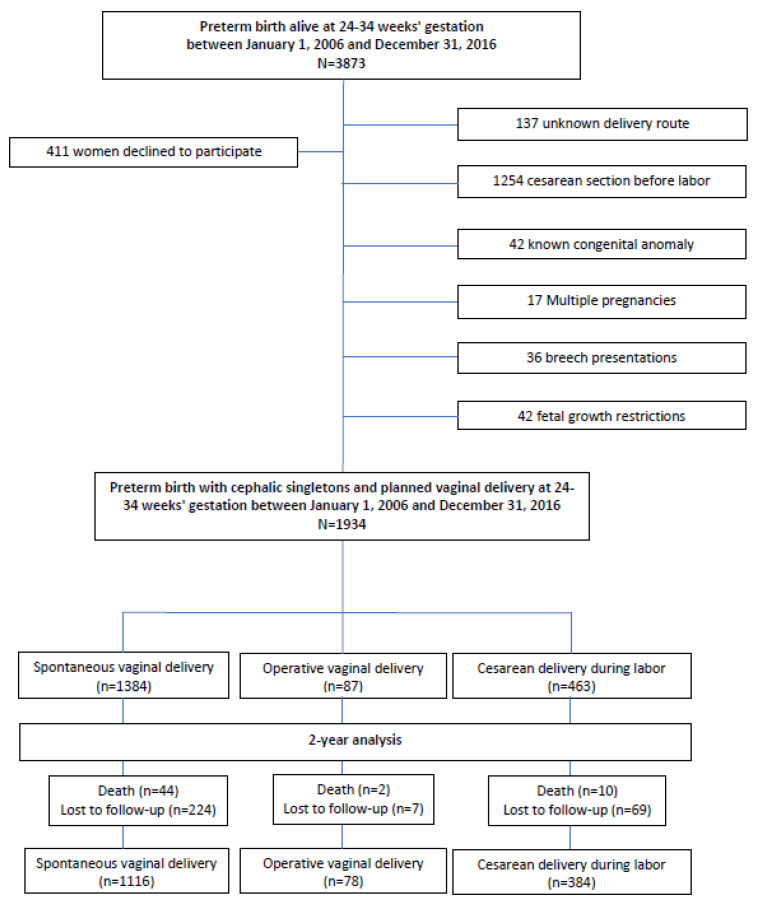
Study flowchart.

Between 1 January 2006 and 31 December 2016, 3873 children were born alive at 24–34 WG in the Pays de la Loire region and were included in the LIFT cohort. After exclusions, 1934 preterm births with cephalic singletons and planned vaginal deliveries at 24–34 WG between 1 January 2006 and 31 December 2016 were included in this study. Of these 1934 deliveries, 1384 (71.6%) included SVD, 87 (4.5%) included OVD, and 463 (23.9%) included CS deliveries during labor (Figure 1); in addition, 12.5% (n = 241) of newborns were born between 25 + 0 and 27 + 6 WG, 34% (n = 657) between 28 + 0 WG and 31 + 6 WG, and 53.6% (n = 1036) between 32 and 33 + 6 WG.

Rates of children who died before hospital discharge were similar between groups (3.2% after SVD, 2.3% after OVD, and 2.2% after CS delivery, *p* = 0.79). The analysis of neurocognitive development at 2 years of corrected age was then carried out on 1578 children (81.6% of the initial cohort): 1116 children after SVD (80.6%), 78 (89.7%) after OVD, and 384 (82.9%) after CS deliveries. Only three (0.1%) children died after discharge and before 2 years old (cause unspecified) after SVD. Of the population who had a follow-up at 2 years, 97.3% (1536/1578) of the children had been examined by a pediatrician and 85.5% (1350/1578) had a completed neurodevelopmental status assessment with an ASQ score.

Table 1 details the maternal and obstetric characteristics according to the mode of delivery, with many missing data due to the fact that the LIFT longitudinal cohort is a neonatal and pediatric follow-up program without exhaustive obstetric data. Nevertheless, the maternal and obstetric characteristics were similar between groups, except for maternal chronic diseases (chronic hypertension and preexisting type 1 or 2 diabetes).

Table 2 details the neonatal characteristics and outcomes according to the mode of delivery. Children born after OVD, compared to SVD or CS during labor, significantly differed with a higher mean gestational age, higher mean birth weight, less frequent Apgar score of <7 at 5 min, and less frequent neonatal procedures in the delivery room (Table 2). Of the 1934 infants included, 329 (17.0%) developed at least one complication during the neonatal period (Table 2). Neonates with SVD, OVD, and CS did not significantly differ in survival without morbidity (82.8% vs. 86.2% vs. 82.7%, respectively; *p* = 0.71).

Among the 1578 children that were followed-up with at 2 years of corrected age, the rate of non-optimal neurodevelopment was 17.7% (n = 279) and did not differ significantly among the three groups SVD, OVD, and CS (18.3%, 18.0%, and 15.9%, respectively, *p* = 0.57) (Table 3). Moreover, the growth characteristics of the children at 2 years of corrected age were similar between groups (head circumference, height, and weight) (Table 3).

Children with non-optimal neurodevelopment at 2 years of corrected age differed according to their maternal socioeconomic status, PPROM longer than 24 h, gestational age at birth, birth weight, Apgar score of <7 at 5 min, lack of spontaneous closure of ductus arteriosus, BPD, and PVL in the neonatal period but not according to OVD compared to SVD and CS (Table 4).

**Figure 2:**
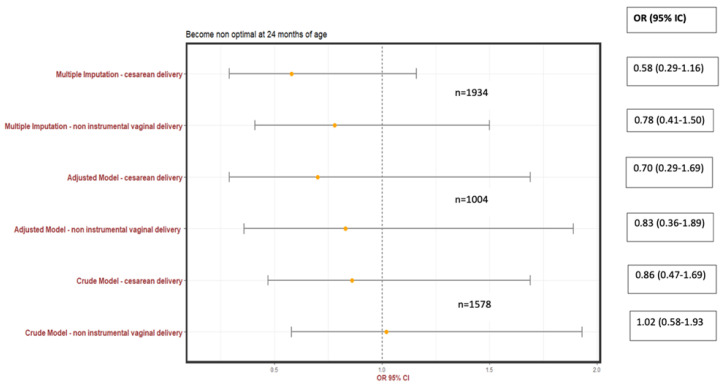
Non-optimal neurodevelopment at 2 years corrected age according to the mode of delivery (reference: operative vaginal delivery).

OVDs for cephalic singletons and planned vaginal deliveries from 24 to 35 WG, compared to SVDs, were not significantly associated with non-optimal neurodevelopment at 2 years of age in either the univariate analysis (Table 5) (crude odds ratio (OR) 1.02; 95% confidence interval (CI) 0.58–1.93) or the multivariable logistic regression analysis adjusted for potential confounders (adjusted OR 0.85; 95% CI 0.35–1.95). The sensitivity analysis based on multiple imputations was also consistent with this primary analysis (adjusted OR −0.24; 95% CI −0.74–0.33) (Table 5, Figure 2). Compared to CSs during labor, OVDs for cephalic singletons and planned vaginal deliveries from 24 to 35 WG were also not significantly associated with non-optimal neurodevelopment at 2 years of age (adjusted OR 0.76; 95% CI 0.31–1.80) (Table 5). The sensitivity analysis based on multiple imputations was also consistent with this primary analysis (adjusted OR −0.54; 95% CI −1.53–0.35) (Table 5, Figure 2).

The instrument used for OVD was only reported for 47.1% (n = 41) of included preterm infants born. Forceps were used in 46.4% (n = 19), vacuum in 26.8% (n = 11), and spatulas in 26.8% (n = 11). The mean gestational ages for OVD were similar according to the instrument used for OVD (31.6 ± 2.9 WG, 32.7 ± 0.8 WG, and 31.4 ± 1.9 WG for forceps, vacuum, and spatula, respectively). No preterm infants born by vacuum had a neurological deficit at 2 years of age, whereas four children born by forceps (21%) and three children born by spatulas (27.3%) had a non-optimal neurological development at 2 years of age.

## 4. Discussion

In our study, operative vaginal delivery was not associated with non-optimal neurocognitive development at the corrected age of 2 years for preterm singleton infants without fetal growth restriction born before 34 WG by planned vaginal delivery who survived at discharge, compared to spontaneous vaginal delivery or compared to cesarean section during labor.

Our findings have potentially important implications. The mode of delivery of preterm newborns has been controversial for decades, as the neonatal outcome depends on many factors. The gestational age at birth, prenatal corticosteroids treatment, and chorioamnionitis are the most important known factors for neonatal outcome. In order to reduce the incidence of peripartum hypoxia associated with prematurity and possible prolonged labor, a practice of elective cesarean delivery was recommended in the 1980s, although there was no medical evidence [14]. A recent retrospective cohort study of 271 singleton pregnancies admitted for preterm labor or preterm premature rupture of membranes between 2010 and 2015 and delivered from 22 to 29 WG reported, after adjusting for nulliparity, delivery year, and fetal presentation at the time of delivery, that cesarean delivery performed for standard obstetrical indications was associated with a decreased risk for death in the delivery room or within 24 h after delivery but was not associated with an improvement in the overall morbidity or mortality [15]. In cases of preterm birth with planned vaginal delivery, the stakes would be, on the one hand, to reduce the time to birth for an intrinsically fragile fetus and, on the other hand, the possibility of protecting the fetal head by limiting its compression in the vaginal canal in preterm birth. Given the lack of evidence and the variation of practice and opinion in this area, the medium- and long-term evolution of preterm children born by OVD is still poorly documented. Our population of OVD in preterm infants born before 34 WG (4.5%) seems to be comparable to the large cohorts of preterm infants in the literature [4,5]. In a study from Swedish national registers of more than 40,000 preterm births before 37 WG, the rate of OVD with vacuum was reported in 5.7% of these births, 3.3% of which were before 34 WG [4]. Moreover, in the literature, the neurological outcome of preterm infants born by OVD is still poorly documented and even more so with long-term neurological evaluation. The main result of our study is in agreement with a recent large population-based cohort analysis of 11,662 preterm newborns (median gestational age 36 WG) of whom 2.3% (n = 267) underwent OVD with vacuum [5]. The authors concluded that OVD in preterm infants was not associated with an increase in pediatric neurological hospitalizations and that the long-term neurological development—up to the age of 18 years—of preterm infants was not unfavorable after OVD [5]. To our knowledge, there are no other studies focusing on the long-term neurological outcome after OVD in preterm infants. Furthermore, the rate of neurological complications during the neonatal period was 4.9% in our cohort, which is close to the rate of 4.5% found in the EPIPAGE-2 study [16].

Our study presents several strengths. First, neurocognitive development at 2 years of corrected age was analyzed rather than survival or neonatal complications. Indeed, developmental delay is now recognized as the main pitfall in children born prematurely [15]. Our main outcome, “non-optimal neurocognitive development at 2 years”, was defined according to criteria recognized and validated in the literature [10,17,18,19]. The clinical examination by an experienced pediatrician was standardized and reproducible, and we reported a high rate of clinical examination of the children by a pediatrician at 2 years in our cohort (97.4%) that reinforces the internal validity of the study. In addition, we used the ASQ questionnaire at 2 years, which is not a gold standard evaluation as a psychomotor test but is the most commonly used and recommended neurodevelopmental screening test completed by parents worldwide [18,20,21,22], and it was validated in French population [11]. Moreover, this questionnaire has been shown as a reliable tool to predict neurologic outcomes with a good validity as compared with the Developmental Quotient score [11] and has been used in very large cohorts such as EPIPAGE-2 [16]. The initial classical ASQ score (ASQ abnormal if one and/or two domains failed) recommended by the University of Oregon’s Center showed a good sensitivity of 0.88 [10], but a rather low specificity of 0.57 [11]. Sices et al. [23] related that certain clinicians use a broader definition of two failed domains on the ASQ, but this definition considerably reduced the sensitivity of the tool to 0.60 (specificity of 0.82). Taken together, these results led us to use ASQ as an overall score. Second, the data are robust: a large number of preterm children were included in the neonatal period (n = 1934) who were followed-up prospectively, with a low rate of being lost to follow-up (15.5%, n = 300). Furthermore, our analysis tried to consider planned vaginal delivery in fetuses without additional pathology in order to avoid confounding factors due to pre-delivery events such as placental insufficiency. Thus, fetuses with fetal growth restriction that could be the consequence of obstetric or maternal pathology were excluded. Our population is therefore made up of a homogeneous group of preterm fetuses with a similar fragility before delivery, which made it possible to compare our three groups (SVD, OVD, and CS during labor), limiting the confounding factors. Third, all included children in the LIFT cohort were cared for by different obstetric and neonatology teams that belonged to the same perinatal network throughout the study period, which especially avoided significant variation regarding the management of pregnancy and labor and need for obstetric intervention at birth (operative vaginal delivery, cesarean delivery, etc.). This allowed for standardized protocols to be followed throughout the study period, thus mitigating differences in outcomes due to variations in clinical practice.

Nevertheless, we must acknowledge some limitations. First, our study was multicentric, thus limiting a recruitment bias, but it included maternity units of different levels of neonatal care. Second, infants who died in the labor ward and before discharge from the hospital were not systematically included, no pediatric follow-up was then set up, and therefore, neonatal mortality could not be analyzed. Nonetheless, we would like to underline that the rates of children who died before hospital discharge were similar between groups. Third, pediatric characteristics were collected very well during study period with few missing data. However, data about maternal and obstetric characteristics were missing before 2012, which should induce some bias. In order to overcome this problem, an analysis after multiple imputations of missing data was carried out and consolidated the results already obtained after logistic regression. Fourth, unfortunately, the neonatal acidemia, duration of labor, duration of the second stage of labor, and indication of OVD were very often missing in the database, which was a prospective, population-based cohort of preterm infants with long-term pediatric objectives, and these obstetric characteristics are covariates that may affect neurodevelopment.

## 5. Conclusions

Our study showed no significant association between operative vaginal delivery and non-optimal neurocognitive development at 2 years of corrected age in surviving preterm infants born with planned vaginal delivery from 24 to 34 weeks’ gestation. The limitations notwithstanding, our study supports the continued use of operative vaginal delivery in appropriately selected population of preterm singleton fetuses. Further analysis by gestational age at birth and type of instrument used would be particularly interesting.

## Figures and Tables

**Table 1:** Maternal and obstetric characteristics according to the mode of delivery (n = 1934).

	Spontaneous Vaginal Delivery n = 1384	Operative Vaginal Delivery n = 87	Cesarean Section during Labor n = 463	*p* Value ^a^
Age, y	29.5 ± 5.5	30.6 ± 4.8	30.7 ± 5.2	0.50
*Missing data*	*56.7%*	*43.7%*	*74.5%*	
BMI before pregnancy, kg/m^2^	23.3 ± 1.1	23.7 ± 0.6	23.7 ± 0.9	0.37
*Missing data*	*87.4%*	*76%*	*93%*	
Tobacco use	47 (23.3)	2 (14.3)	18 (24.0)	0.72
*Missing data*	*85.4%*	*83.9%*	*83.8%*	
ART	44 (8.8)	5 (14.7)	19 (12.6)	0.24
*Missing data*	*73.7%*	*60.9%*	*67.4%*	
Chronic hypertension	35 (13.5)	3 (13.6)	50 (61.7)	<0.001
*Missing data*	*81.3%*	*74.7%*	*82.5%*	
Preexisting type 1 or 2 diabetes	1 (0.4)	3 (14.3)	0	<0.001
*Missing data*	*83%*	*76%*	*93%*	
Maternal socioeconomic status				0.20
Low	102 (12.9)	2 (3.4)	32 (12.3)	
Intermediate	574 (72.5)	47 (79.7)	197 (75.8)	
High	116 (14.6)	10 (16.9)	31 (11.9)	
*Missing data*	*42.8%*	*32.2%*	*32.3%*	
Paternal socioeconomic status				0.32
Low	102 (12.7)	2 (3.4)	32 (11.8)	
Intermediate	560 (69.7)	45 (76.3)	193 (71.2)	
High	141 (17.6)	12 (20.3)	46 (17.0)	
*Missing data*	*42.0%*	*32.2%*	*29.4%*	
Threatened preterm birth	980 (70.8)	57 (65.5)	248 (53.6)	0.11
Gestational diabetes mellitus	16 (1.2)	4 (4.6)	3 (0.6)	0.99
Corticosteroid treatment	796 (57.5)	53 (60.9)	256 (55.3)	0.54
Magnesium sulfate	49 (3.5)	4 (4.6)	11 (2.4)	0.38
IOL	44 (3.2)	1 (1.1)	19 (4.1)	0.32

Values are given as mean ± SD or number (percentage) unless otherwise indicated. *BMI*, body mass index; *ART*, assisted reproductive technology; *IOL*, induction of labor. ^a^ Categorical variables were compared with c2 tests and continuous ones with Wilcoxon tests.

**Table 2:** Neonatal characteristics and outcomes according to the mode of delivery (n = 1934).

	Spontaneous Vaginal Delivery n = 1384	Operative Vaginal Delivery n = 87	Cesarean Section During Labor n = 463	*p* Value ^a^
Gestational age at birth, weeks	30.6 ± 2.5	31.7 ± 1.9	30.6 ± 2.3	0.001
25–27 WG	189 (13.7)	3 (3.4)	49 (10.6)	
28–31 WG	448 (32.4)	22 (25.3)	187 (40.4)	
32–34 WG	747 (54.0)	62 (71.3)	227 (49.0)	
Level of neonatal care				0.03
Maternity with NICU	1292 (93.5)	83 (95.4)	449 (97.0)	
Maternity without NICU	59 (4.3)	4 (4.6)	13 (2.8)	
Delivery at home	28 (2.0)	0	0	
*Missing data*	*0.4%*	*0*	*0.2%*	
PPROM > 24 h	348 (25.1)	20 (23.0)	100 (21.6)	0.29
Chorioamnionitis	76 (5.5)	3 (3.4)	30 (6.5)	0.48
Birth weight, g	1653 ± 481	1807 ± 395	1564 ± 445	<0.001
Z-score of birth weight	0.29 ± 0.80	0.14 ± 0.73	0.02 ± 0.86	<0.001
<−1 SD	47 (3.4)	5 (5.7)	47 (10.2)	
Between −1 SD and 0 SD	451 (32.7)	28 (32.2)	184 (39.7)	
Between 0 and +1 SD	658 (47.7)	44 (50.6)	183 (39.5)	
>+1 SD	223 (16.2)	10 (11.5)	49 (10.6)	
Male	793 (57.3)	61 (70.1)	272 (58.7)	0.06
Height at birth, cm	40.0 ± 1.1	42.0 ± 0.8	39.6 ± 1.8	0.15
Head circumference at birth, cm	28.3 ± 2.1	29.4 ± 1.8	28.3 ± 2.2	0.22
Apgar score < 7 at 5 min	104 (7.5)	4 (4.6)	61 (13.2)	<0.001
Neonatal procedures in delivery room	960 (69.4)	20 (23.0)	85 (18.4)	<0.001
Mask ventilation	512 (39.4)	50 (61.0)	190 (45.1)	
Intubation	38 (22.9)	11 (13.4)	127 (30.2)	
External cardiac massage	324 (5.0)	1 (1.2)	19 (4.5)	
Intraventricular hemorrhage ≥ grade 3	31 (2.2)	2 (2.3)	14 (3.0)	0.64
Periventricular leukomalacia	36 (2.6)	3 (3.4)	13 (2.8)	0.88
Bronchopulmonary dysplasia	56 (4.1)	2 (2.3)	15 (3.2)	0.56
Need closure of ductus arteriosus	174 (12.6)	9 (10.3)	52 (11.2)	0.65
Necrotizing enterocolitis	16 (1.2)	1 (1.1)	6 (1.3)	0.97
Neonatal morbidity ^b^	237 (17.2)	12 (13.8)	80 (17.3)	0.71
Neonatal death	44 (3.2)	2 (2.3)	10 (2.2)	0.79

Values are given as mean ± SD or number (percentage) unless otherwise indicated. WG, weeks of gestation; *NICU*, neonatal intensive care unit; *PPROM*, preterm premature rupture of membranes. ^a^ Categorical variables were compared with c2 tests and continuous ones with Wilcoxon tests; ^b^ Neonatal morbidity was a composite variable, defined by at least one of the following criteria: severe intraventricular hemorrhage ≥ grade 3, periventricular leukomalacia, bronchopulmonary dysplasia, persistence of the ductus arteriosus with necessary medical and/or surgical treatment, and necrotizing enterocolitis.

**Table 3:** Pediatric and neurological outcomes at 2 years of corrected age according to the mode of delivery (n = 1578).

	Spontaneous Vaginal Delivery n = 1384	Operative Vaginal Delivery n = 87	Cesarean Section During Labor n = 463	*p* Value ^a^
Pediatric death (<2 years)	3 (0.2)	0	0	-
Loss of follow-up	224 (16.2)	7 (8.0)	69 (14.9)	0.05
**At 2 years of corrected age**	**n = 1116 (80.6)**	**n = 78 (89.7)**	**n = 384 (82.9)**	
Height, cm	87.5 ± 3.8	87.5 ± 4.0	87.3 ± 5.3	
Weight, kg	12.1 ± 1.6	12.2 ± 1.3	12.1 ± 2.5	
Head circumference, cm	48.8 ± 1.8	49.4 ± 1.8	48.7 ± 1.7	
Non-optimal clinical examination	188 (16.8)	13 (16.7)	56 (14.6)	0.51
*Missing data*	*2.8%*	*2.6%*	*2.3%*	
ASQ score	244.7 ± 38.4	246.1 ± 31.9	242.4 ± 36.3	0.58
Non-optimal ASQ score (<185)	54 (5.7)	7 (2.9)	19 (5.8)	0.61
*Missing data*	*14.9%*	*15.1%*	*15.1%*	
**Primary outcome ^b^**	**204 (18.3)**	**14 (18.0)**	**61 (15.9)**	**0.57**

Values are given as mean ± SD or number (percentage) unless otherwise indicated. ^a^ Categorical variables were compared with c2 tests and continuous ones with Wilcoxon tests; ^b^ primary outcome was non-optimal neurocognitive development at 2 years corrected age that included children with non-optimal clinical examination and/or non-optimal psychomotor development according to ASQ scale.

**Table 4:** Maternal, pregnancy and labor characteristics, and neonatal outcome according to neurocognitive development at 2 years corrected age (n = 1578).

	Non-Optimal Neurocognitive Development ^a^ n = 279	Optimal Neurocognitive Development n = 1299	*p* Value ^b^
Chronic hypertension	14 (5.0)	63 (4.8)	1.0
Maternal socioeconomic status			0.004
Low	32 (19.3)	94 (10.3)	
Intermediate	114 (68.7)	690 (75.2)	
High	20 (12.0)	133 (14.5)	
*Missing data*	*40.5%*	*29.4%*	
PPROM > 24 h	51 (18.3)	326 (25.1)	0.02
*Missing data*	*1.1%*	*4.0%*	
Chorioamnionitis	16 (5.8)	65 (5.0)	0.72
*Missing data*	*1.1%*	*0.2%*	
Magnesium sulfate treatment	11 (3.9)	38 (2.9)	0.48
*Missing data*	*4.7%*	*3.5%*	
Corticosteroid treatment	155 (55.6)	739 (56.9)	0.73
*Missing data*	0.7%	0.4%	
IOL	9 (3.2)	45 (3.5)	0.99
*Missing data*	*0.4%*	*2.0%*	
Gestational age at birth, weeks	29.7 (2.7)	30.9 (2.3)	<0.001
25–27 WG	62 (22.2)	113 (8.7)	<0.001
28–31 WG	117 (41.9)	438 (33.7)	
32–34 WG	100 (35.9)	748 (57.6)	
Mode of delivery			0.57
Spontaneous vaginal delivery	204 (73.1)	912 (70.2)	
Operative vaginal delivery	14 (5.0)	64 (4.9)	
Cesarean section during labor	61 (21.9)	323 (24.9)	
Birth weight, g	1475 ± 489	1685 ± 447	<0.001
Z-score of birth weight	0.20 ± 0.8	0.23 ± 0.8	0.61
<−1 SD	15 (5.4)	66 (5.1)	
Between −1 SD and 0 SD	97 (34.9)	436 (33.6)	
Between 0 and +1 SD	127 (45.7)	605 (46.6)	
>+1 SD	39 (14.0)	190 (14.6)	
Level of neonatal care			
Maternity with NICU	259 (92.8)	1237 (95.2)	0.29
Maternity without NICU	14 (5.0)	49 (3.8)	
Delivery at home	6 (2.2)	13 (1.0)	
Apgar score < 7 at 5 min	38 (13.6)	87 (6.7)	<0.001
Neonatal procedures in delivery room			<0.001
Mask ventilation	90 (35.4)	542 (45.1)	
Intubation	93 (36.6)	262 (21.8)	
External cardiac massage	14 (5.5)	27 (2.2)	
Need closure of ductus arteriosus	65 (23.3)	112 (8.6)	<0.001
*Missing data*	*0*	*0*	
Bronchopulmonary dysplasia	19 (6.8)	37 (2.9)	0.002
*Missing data*	*0.4%*	*0.2%*	
Intraventricular hemorrhage ≥ grade 3	6 (2.2)	13 (1.0)	0.19
*Missing data*	*0*	*0*	
Periventricular leukomalacia	27 (9.7)	13 (1.0)	<0.001
*Missing data*	*0*	*0*	
Necrotizing enterocolitis	6 (2.2)	12 (0.9)	0.15
*Missing data*	*0*	*0*	
Neonatal morbidity ^c^	91 (32.7)	157 (12.1)	<0.001
*Missing data*	*0.4%*	*0.1%*	

Values are given as mean ± SD or number (percentage) unless otherwise indicated. *PPROM*, preterm premature rupture of membranes; IOL, induction of labor; *WG*, weeks of gestation; *NICU*, neonatal intensive care unit. ^a^ Non-optimal neurocognitive development at 2 years corrected age included children with non-optimal clinical examination and/or non-optimal psychomotor development according to ASQ scale; ^b^ categorical variables were compared with c2 tests and continuous ones with Wilcoxon tests; ^c^ neonatal morbidity was a composite variable, defined by at least one of the following criteria: severe intraventricular hemorrhage ≥ grade 3, periventricular leukomalacia, bronchopulmonary dysplasia, persistence of the ductus arteriosus with necessary medical and/or surgical treatment, and necrotizing enterocolitis.

**Table 5:** Univariate and multivariate analysis of non-optimal neurodevelopment at 2 years corrected age (n = 1578).

Variable	Crude OR (95% CI)	*p*-Value	Adj. OR (95% CI)	*p*-Value	Imp. OR (95% CI)	*p* Value
Male	1.18 (0.90–1.54)	0.23				
Gestational age at birth	0.82 (0.78–0.87)	<0.001	-	-		
≤27 WG	4.10 (2.82–5.96)	<0.001	1.28 (0.50–3.25)	0.60		
28–31 WG	2.00 (1.49–2.68)	<0.001	1.33 (0.75–2.37)	0.33		
32–34 WG	1.00	-	1.00	-		
Birth weight	1.00 (1.00–1.00)	<0.001	1.00 (0.99–1.00)	0.45		
Z-score of birth weight (SD)						
<−1	1.00	-				
Between −1 and 0	0.98 (0.54–1.82)	0.94				
Between 0 and +1	0.92 (0.58–1.91)	0.79				
>+1	0.90 (0.56–2.10)	0.76				
Maternal socioeconomic status						
Low	1.00	-	1.00	-	1.00	-
Intermediate	0.49 (0.31–0.77)	0.002	0.54 (0.34–0.88)	0.01	−0.68 (−3.22–0.61)	<0.01
High	0.44 (0.24–0.81)	0.01	0.47 (0.24–0.92)	0.03	−0.79 (−2.51–0.31)	<0.01
Chronic hypertension	1.04 (0.55–1.82)	0.91				
PPROM > 24 h						
No	1.00	-	1.00	-	1.00	-
Yes	0.67 (1.07–2.08)	0.02	0.64 (0.41–0.99)	0.05	−0.41 (0.18–2.27)	0.02
Chorioamnionitis						
No	1.00	-				
Yes	1.15 (0.64–1.98)	0.61				
Magnesium sulfate						
No	1.00	-				
Yes	1.36 (0.66–2.61)	0.38				
Corticosteroid treatment						
No	1.00	-				
Yes	0.95 (0.73–1.23)	0.68				
Induction of labor						
No	1.00	-				
Yes	0.93 (0.42–1.83)	0.84				
Level of maternity						
Without NICU	1.00	-				
With NICU	0.74 (0.41–1.42)	0.34				
At home	1.62 (0.49–4.93)	0.41				
Apgar score < 7 at 5 min	2.20 (1.45–3.27)	<0.001	1.29 (0.70–2.37)	0.41		
Immediate neonatal procedures						
No procedure	1.00	-	1.00	-	1.00	-
Mask ventilation	1.08 (0.76–1.56)	0.66	1.18 (0.72–1.93)	0.50	−0.07 (0.18–0.36)	0.72
Intubation	2.32 (1.61–3.35)	<0.001	1.99 (1.12–3.54)	0.02	0.79 (0.18–4.37)	<0.001
External cardiac massage	3.98 (1.64–6.75)	<0.001	2.43 (0.90–6.54)	0.08	1.24 (0.35–3.50)	<0.001
Need to close the ductus arteriosus						
No	1.00	-	1.00	-		
Yes	3.22 (2.29–4.50)	<0.001	1.03 (0.34–3.06)	0.96		
BPD						
No	1.00	-	1.00	-		
Yes	2.50 (1.39–4.36)	0.002	1.37 (0.57–3.26)	0.48		
IVH grade III or IV						
No	1.00	-				
Yes	2.17 (0.76–5.55)	0.12				
PVL						
No	1.00	-	1.00	-	1.00	-
Yes	10.6 (5.50–21.48)	<0.001	5.47 (1.43–20.81)	0.01	2.20 (0.35–6.35)	<0.001
NEC						
No	1.00	-				
Yes	2.36 (0.81–6.13)	0.09				
Neonatal morbidity ^a^						
No	1.00	-	1.00	-		
Yes	3.54 (2.61–4.77)	<0.001	1.34 (0.45–3.96)	0.59		
SVD (versus OVD)	1.02 (0.58–1.93)	0.94	0.85 (0.37–1.95)	0.70	−0.24 (−0.74–0.33)	0.46
CS (versus OVD)	0.86 (0.47–1.69)	0.65	0.76 (0.31–1.80)	0.53	−0.54 (−1.53–0.35)	0.12

Unadjusted and adjusted logistic regression analyses. All the variables in the table were incorporated in the multivariable logistic models. *WG*, weeks of gestation; *CI*, confidence interval; *OR*, odds ratio; *PPROM*, preterm premature rupture of membranes; *NICU*, neonatal intensive care unit; *BPD*, bronchopulmonary dysplasia; *IVH*, intraventricular hemorrhage; *PVL*, periventricular leukomalacia; *NEC*, necrotizing enterocolitis; SVD, spontaneous vaginal delivery; OVD, operative vaginal delivery; CS, cesarean section during labor. ^a^ Neonatal morbidity was a composite variable, defined by at least one of the following criteria: severe IVH grade 3 or 4, PVL, BPD, persistence of the ductus arteriosus with necessary medical and/or surgical treatment, and NEC.

## Data Availability

The data presented in this study are available on request from the corresponding author. The data are not publicly available due to institutional policy.
